# Abstracts/Sommaires/Zusammenfassungen

**DOI:** 10.1155/S1463924679000377

**Published:** 1979


### Abstracts Sommaires Zusammenfassungen

### Page 125

**Teaching a graduate level course in automatic chemical analysis.**

Richard F. Browner

A brief description and course outline is given for a graduate level course in automatic chemical analysis taught at the Georgia Institute of Technology. The course is presented in an economic and managerial context, in contrast to the more usual background of electronics and computing, although these aspects are considered as an important part of the course. The purpose of the course is to alert the students to the potential of automatic procedures in their later careers. A wide range of topics is covered and great emphasis is placed on the co-ordinated approach that must be adopted in a large laboratory as new automatic procedures replace older manual methods. The specific example of gas liquid chromatography is cited to illustrate the approach.

**Enseignement d'un cours de chimie analytique automatique pour étudiants gradués**

Une brève description d'un cours de chimie analytique automatique pour étudiants gradués au Georgia Institut of Technology est donnée. Le cours est présenté dans un contexte economique at administratif, faisant contraste aux aspects habituels et tels que l'electronique et la programmation, qui sont néanmonis bien importants pour ce cours. Le but de ce cours est de sensibiler les etudiants pour les possibiltés de l'automation en une de leurs futurs emplois. Une grande varieté de sujets est discutée et on appuye sur l'importance d'une approche coordonnée dans un grand laboratoire où les nouveaux systemes automatiques doivent remplacer les anciennes methodes manuelles. L'example specifique de la chromatographie en phase gazeuse est utilisé pour illustrer cet approche.

**Studenten-Lehrgang fuer Automation in der chemischen Analytik**

Es wird eine kurze Beschreibung und ein Kursprogramm dargelegt für einen Studentenlehrgang in Automation in der chemischen Analytik, der am Georgia Institute of Technology unterrichtet wird. Der Lehrgang wird in einem okonomischen und betriebswirtschaftlichen Zusammenhang offeriert. Das steht im Gegensatz zu den ublicheren Aspekten von Elektronik und Programmierung, obwohl diese als ein wichtiger Teil des Lehrgangs betrachtet werden. Ziel des Kurses ist, die Studenten für Ihre spätere Karriere auf das Potential automatischer Abläufe aufmerksam zu machen. Ein breites Spektrum vòn Themen wird besprochen und mit grossem Nachdruck ein koordiniertes Vorgehen betont, das in grossen Laboratorien gewählt werden muss, wo neue automatische Verfahren ältere manuelle Methoden ersetzen. Dieses Vorgehen wird am spezifischen Beispiel der Gas-Flussig-Chromatographie illustriert.

### Page 128

**Advanced software concepts for employing microcomputers in the laboratory.**

Scott B. Tilden, M. Bonner Denton.

Current trends in the computerisation of specialised custom chemical instrumentation indicate the increasing utilisation of dedicated microprocessors. Conventional software techniques often possess serious limitations in regard to initial development effort, execution speed, flexibility, and/or hardware required. A high-level 'interpretive compiler' software package, convers, is described which offers numerous advantages compared to conventional approaches including high speed operation, high level I/Q, language flexibility, superior memory efficiency and a variety of other highly desirable characteristics.

**Des concepts avances du "software" pour l'utilisation des microordinateurs dans le laboratoire**

Les tendance actuelles de l'instrumentation chimique ordinaire et specialisée dans le domaine des ordinateurs indiquent l'utilisation croissante des microprocesseur dediés. Les techniques conventionelles du "software" ont souvent des limitations graves en ce qui concerne le premier effort de développement, la vitesse d'execution, la flexibilité et/ou le "hardware" exigé. Un "software package" interpretitif et de haut-niveau est décrit qui fournissent de nombreux avantages en comparison des approches conventionelles, y compris l'opération à haute vitesse, l'affluence et le débit de niveau élevé, la flexibilité de langue l'efficacité de souvenir supérieure et une variété d'autres caractèristiques désirables.

**Fortschrittliches Software-Konzept für den Einsatz von Mikroprozessoren im Labor.**

Der derzeitige Trend in der Computerisierung von spezieller, kundenorientierter chemische Instrumentierung zeigt den anwachsenden Einsatz von Mikroprozessoren. Konventionelle Software-Techniken sind oft stark einschränkend in Bezug auf den Anfangs-Entwicklungsaufwand, Rechenzeit, Flexibilität und/oder benötigte Hardware. Ein "high-level interpretive compiler" Software-Paket, konversationell, wird beschreiben, das viele Vorteile bietet im Vergleich zu konventionellen Ansätzen. Diese Vorteile umfassen kurze Rechenzeit, "high level" Ein- und Aus-gabe, Sprachenflexibilität überlegene Speichereffizienz und eine Reihe von anderen wünschbaren Eigenschaften.

### Page 134

**The use of a microcomputer for flexible automation of a liquid chromatograph**

A.D. Mills, I. Mackenzie, R.J. Dolphin

This paper describes the construction and performance of an automatic liquid chromatograph under the control of a microcomputer based on the Intel 8080 microprocessor. The aspects of liquid chromatography which can be enhanced by automation are identified and it is shown that this design provides simple and effective solutions to the problems of flow control, automatic sample handling, and data analysis. The flexibility of software control

**L'utilisation d'un microordinator pour l'automation flexible d'un chromatographe à phase liquide.**

Cet article décrit la construction et le fonctionnement d'un chromatographe à phase liquide sous le contrôle d'un microordinateur fondé sur le microprocesseur Intel 8080. Les aspects de la chromatographie liquide qui peuvent être ameliorés par l'automation sont identifiés. On demontre que le dessein peut fournir des solutions simples et efficaces aux problèmes du contrôl du debit, du traitement automatiques des échantillons et du traitement

**Einsatz eines Mikrocomputers fuer flexible Automation eines Fluessig-Chromatographen**

Diese Arbeit beschreibt die Konstruktion und Leistungen eines automatischen Flussig-Chromatographen der von einem Mikrocomputer mit Intel 8080 Mikroprozessor gesteuert wird. Diejenigen Gebiete in der Flussigchromatographie, wo durch Automation besondere Verbesserungen erzielt werden können, werden identifiziert, und es wird gezeigt, dass diese Konstruktionsart einfache und wirkungsvolle Lösungen auf die Probleme der Durchflussregelung, autoenables the operator to choose from a variety of operational modes and to interact with the instrument in order to achieve optimum performance.

des donnees. La flexibilité du "software" permet a l'opérateur de choisir parmi un grand nombre de modes opérationnels d'une facon interactive avec l'instrument à fin d'obtenir une performance optimale.

matische Probenbehändigung, und Daten-Analyse ermöglicht. Die Flexibilität der Steuerung durch Software ermöglicht der Bedienungsperson eine Reihe von Betriebsarten anzuwahlen und auf das Instrument Einfluss zu nehmen, um eine optimale Leistung zu erzielen.

**Page 140**

**An evaluation of the Kem-O-Mat programmable discrete analyser**

Geoffrey C. Seymour

The Kem-O-Mat is a single channel discrete analyser which is operated under control from a programmable calculator. The programmes can either be supplied by the instrument manufacturer or developed by the operator thereby facilitating the use of the laboratory's own methods on the instrument. The volumes of sample and reagents required for the chemistries are small, thus affording a net saving in running costs. The instrument has been well received by the laboratory staff for several reasons; mainly, simplicity of operation, ease of maintenance, versatility, fast through-put and improved performance with regard to precision in the author's laboratory.

**Evaluation de l'analyseur programmable discontinu Kem-O-Mat**

Le Kem-O-Mat est un analyseur discontinu à un canal qui fonctionne sous le contrôle d'un calculateur programmable. Les programmes peuvent être livrés soit par le producteur soit developpés par l'utilisateur pour meilleur adaption des méthodes propres a son laboratoire. Les volumes des échantillons et des reactifs requis sont bas, ainsi ils reduisent les frais courants. L'instrument a été bien reçu par le personnel du laboratoire pour les raisons suivantes: simple opération, peu maintien, sa faculté d'adaptation , son grand débit et pour les resultats plus precis obtenus dans le laboratoire de l'auteur.

**Eine Beurteilung des programmierbaren, diskontinuierlichen Kem-O-Mat Analysators**

Der Kem-O-Mat ist eindiskoantinuierlicher Einkanalanalysator der von einem programmierbaren Rechner gesteuert wird. Die Programme konnen entweder vom Instrumentenhersteller geliefert werden oder aber können durch die Bedienungsperson entwickelt werden, womit die Benutzung der im Laboratorium verwendeten Methoden auf dem Instrument erleichtert wird. Die für die Chemie benötigten Volumina vol Probe und Reagenzien sind klein, was eine Senkung der Betriebskosten erlaubt. Das Instrument wurde vom Laboratoriumspersonal gut akzeptiert aus den folgenden Gründen: Einfachheit der Arbeitsweise, leichter Unterhalt, Vielseitigkeit, hoher Durchsatz und verbesserte Leistungen. im Bezug auf Präzision im Laboratorium des Autors.

### Notes for Contributors

### Presentation of manuscripts

Manuscripts should be typed (double-spaced) on one side of the paper only and with generous margins. The title should be brief and informative avoiding the word "new" and its synonyms. The full list of authors with their affiliations and full address(es) should appear on the title page. On a separate sheet an abstract of no more than 150 words is required. This should succinctly describe the scope of the contribution and highlight significant findings or innovations. It should be written in a style which can easily be translated into French and German.

*The Concise Oxford Dictionary* and Fowler's *Modern English Usage* (both published by Oxford University Press) should be used as the standard for spelling and grammar. Abbreviations should be limited to those generally recognised, or where a frequently occuring term is abbreviated it should, in the first instance, be explained thus "flow injection analysis (FIA) ..." and the abbreviation used thereafter. Abbreviations, for standard measures and units should follow SI recommendations. There are various publications giving guidance on the use of SI units.

References should be indicated in the text by numerals following the author's name, i.e. Skeggs [6]. On a separate sheet of paper, list all references in numerical order thus:

[6] Skeggs, L.T., *American Journal of Clinical Pathology,* 1959, **28**, 311.

Note that journal titles are given in full. Where there is more than one author, the form Foreman et al. should be used in the text but all authors should be named in the list of references. When reference is made to a chapter in a book the reference should take the following form:

[7] Malmstadt, H.V. in "Topics in Automatic Chemistry" Ed. Stockwell P.B. and Foreman J.K. 1978 Horwood, Chichester, pp. 68-70.

Only work which has been published or has been accepted for publication should be cited. Avoid the citation of documents which are subject to restricted circulation, patent literature, unpublished work and personal communications. The latter can be mentioned in the text in parenthesis.

To illustrate a paper line diagrams are preferred to photographs. Photographs should only be used when they significantly add to the discussion. Diagrams, charts and graphs should be carefully drawn in black ink on stout card or heavy quality tracing paper. Most illustrations are reduced for publication; to allow for this originals should be between 16 and 36 cm wide (the depth must not exceed 50 cm). The lettering of diagrams should be sufficiently clear to withstand reduction. Except in the case of proper names, all lettering should be in lower case print. If photographs are used they must be supplied in the form of clear, unmounted, glossy, black and white prints. "Instant" photographs are not normally acceptable. All illustrations must be identified on the reverse showing the figure number and the author's name.

Each illustration should have a fully explanitory caption. Captions should be typed together on a separate sheet of paper; they must *not* be an inseparable part of the illustration.

